# Comparison of Knee Stability, Strength Deficits, and Functional Score in Primary and Revision Anterior Cruciate Ligament Reconstructed Knees

**DOI:** 10.1038/s41598-018-27595-8

**Published:** 2018-06-15

**Authors:** Do Kyung Kim, Geon Park, Kamarulzaman Bin Haji M. S. Kadir, Liang-Tseng Kuo, Won Hah Park

**Affiliations:** 1 Department of Sports Medicine, Samsung Medical Center, Sungkyunkwan University School of Medicine, Seoul, Korea; 2 Department of Orthopaedic Surgery, Hospital Tengku Ampuan Rahimah Klang, Ministry of Health, Selangor, Malaysia; 3 Department of Orthopaedic Surgery and Sports Medicine Center, Chang Gung Memorial Hospital, Chiayi, Taiwan

## Abstract

Comparing to primary surgery, revision ACL reconstruction is more technically demanding and has a higher failure rate. Theoretically, rehabilitation can improve knee function after ACL reconstruction surgery. This study aimed to compare knee stability, strength, and function between primary and revision ACL reconstructed knees. 40 primary and 40 revision ACL reconstruction surgeries were included between April 2013 and May 2016. Patients with revision surgery had a higher anteroposterior translation comparing those with primary reconstruction (median laxity, 2.0 mm vs. 3.0 mm, *p* = 0.0022). No differences were noted in knee extensor at 60°/sec or 180°/sec (*p* = 0.308, *p* = 0.931, respectively) or in flexor muscle strength at 60°/sec or 180°/sec between primary and revision ACL reconstruction knees (*p* = 0.091, *p* = 0.343, respectively). There were also no significant differences between functional scores including IKDC score and Lysholm score in primary versus revision surgeries at 12th months after index operation (*p* = 0.154, *p* = 0.324, respectively). In conclusion, despite having higher anteroposterior instability, patients with revision ACL reconstruction can have non-inferior outcomes in isokinetic knee strength and function compared to those with primary ACL reconstruction after proper rehabilitation.

## Introduction

Anterior cruciate ligament reconstruction (ACLR) surgery is a common procedure to improve functional stability after an anterior cruciate ligament (ACL) injury. The successful rate of primary ACLR is approximated to be 75% to 97% among patients^1,2^. This means that almost one-fourth of primary ACLR surgeries bound to fail despite improved surgical methods^3^. The causes associated with failed ACLR surgery are surgical factors and repetitive injury to the knee^4,5^. Among these patients, approximately 2.9 to 8.9% underwent the following revision ACLR surgery^6,7^.

Compared to primary ACLR surgery, revision surgery is more technically demanding. In revision surgery, accessibility to original tunnels is a key factor of success since the removal of hardware is often required and bone grafting is likely to be necessary^8^. This contributes to an estimated 35% failure rate of revision ACLR surgery, which translated to approximately 54% of patients returning to pre-injury levels^9^. Therefore, with these technical difficulties, the success rate of revision ACLR surgery is commonly less compared to primary ACLR surgery^10^.

In addition to regain the joint stability, the goal of ACLR surgery is also to recover the function of the knee joint and muscle strength^10^. To maximize the outcomes of surgery and enhance the functional recovery, an appropriate rehabilitation program is incorporated into the routine postoperative care. The impaired knee functions associated with ACL injury include instability in dynamic movement and quadriceps weakness. As being critical to dynamic joint stability, the quadriceps muscle weakness eventually leads to decreased knee function, and poor exercise performance, and may contribute early onset of osteoarthritis^11–13^. Thus, the strength of muscle around the knee joint, especially the quadriceps muscle, was the main target of training and keep indicator for monitoring the functional recovery after ACLR surgery^13^.

Thus, to evaluate the success of primary ACLR surgery, joint stability, muscle strength and knee function were common outcomes reported in previous studies^1,10,11^. However, there were limited studies which have assessed muscle strength recovery following revision ACLR surgery. Thus, we performed this study to assess the stability and functional recovery after primary and revision ACLR surgery under the scheduled rehabilitation program.

This study aimed to (1) compare recovery of knee extensor muscle (quadriceps) and flexor muscle (hamstring) strength in primary versus revision ACLR surgery, and (2) compare clinical functional and stability outcomes in primary versus revision ACLR surgery.

## Results

### Demographics

There were 80 male participants included in this study. There were no statistically significant differences in age, height and body weight between primary and revision groups (*p* = 0.538, *p* = 0.105, *p* = 0.969 respectively) (Table 1).

**Table 1:** Demographic characteristics of study subjects.

	Primary	Revision	*p* value
Group size (n)	40	40	
Age (years)	31 (23–35)	29.5 (24–38)	0.538
Height (cm)	173.6 (171.1–176.0)	171.2 (170.0–174.6)	0.105
Weight (kg)	76.3 (72.1–81.7)	76.3 (71.7–81.5)	0.969

Results are shown as median with interquartile range.

### Ligament stability

**Figure 1:**
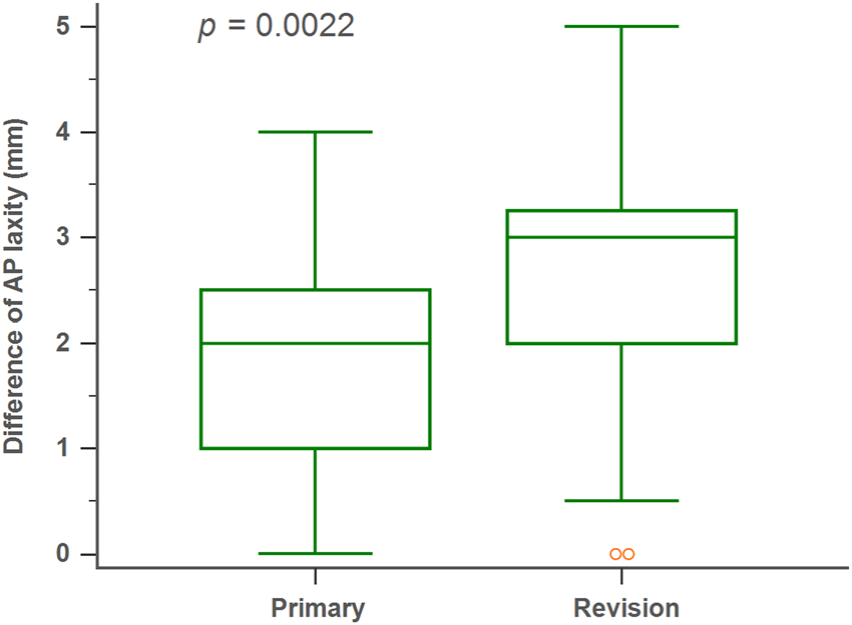
Box-and-whisker plot for anteroposterior laxity in the primary and the revision ACL reconstruction groups. The revision ACLR group had greater instability comparing with primary group. AP anteroposterior.

The AP laxity between groups was statistically different (*p* = 0.0022) (Fig. 1). The median laxity was 2.0 mm (IQR, 1.0 mm–2.5 mm) in primary group and 3.0 mm (IQR, 2.0 mm–3.25 mm) in revision group. AP laxity between involved and healthy limb was also recorded. For the primary group, the difference was <3 mm in 30 cases, 3–5 mm in 10 cases, and >5 mm in none of the cases. For the revision group. The difference was <3 mm in 7 cases, 3–5 mm in 28 cases, and >5 mm in 5 cases. The degree of side-to-side laxity difference was significantly different between two groups (*X*^2^ test, *p* < 0.0001) (Table 2).

**Table 2:** Anteroposterior ligament laxity.

Laxity	Primary, n (%)	Revision, n (%)
<3 mm	30 (75.0%)	7 (17.5%)
3–5 mm	10 (25.0%)	28 (70.0%)
>5 mm	0 (0%)	5 (12.5%)

Results are shown as number with percentage.

Chi-Squared test: *p* < 0.0001.

### Isokinetic knee strength

Deficits in knee extensor strength were not significantly different between primary and revision ACL reconstruction groups, showing 18.0% (IQR, 13.0–28.0%) and 22.0% (IQR, 14.0–33.0%) respectively, at 60°/sec. The knee extensor also showed 17.0% (IQR, 7.0–24.5%) and 14.5% (IQR, 10.0–21.5%) deficit in primary and revision group, respectively at 180°/sec. Moreover, there were no differences in knee flexor strength between primary and revision ACLR groups, showing 9.5% (IQR, 1.5–14.5%) and 12.5 (IQR, 6.0–21.5%), respectively, at 60°/sec and 4.5% (IQR, −2.0–16.5%) and 11.5% (IQR, −0.5–15.0%), respectively, at 180°/sec (Table 3).

**Table 3:** Isokinetic knee strength deficits.

Isokinetic strength deficits	Primary (%)	Revision (%)	*p* value
Extensor 60°/sec	18.0 (13.0–28.0)	22.0 (14.0–33.0)	0.308
Extensor 180°/sec	17.0 (7.0–24.5)	14.5 (10.0–21.5)	0.931
Flexor 60°/sec	9.5 (1.5–14.5)	12.5 (6.0–21.5)	0.091
Flexor 180°/sec	4.5 (−2.0–16.5)	11.5 (−0.5–15.0)	0.343

Results are shown as median with interquartile range.

### Knee functional score

For primary reconstruction, the median IKDC subject score was 81.05 (IQR, 71.7–89.7) and the median Lysholm score was 95.0 (IQR, 89.0–99.0) at the last follow-up. For the revision reconstruction group, the median IKDC subject score was 80.4 (IQR, 67.85–83.85) and the median Lysholm score was 92.5 (IQR, 88.0–95.0) at the last follow-up. There were no differences in functional scores including IKDC score and Lysholm score for primary reconstruction versus revision surgery (*p* = 0.154, *p* = 0.324, respectively) (Table 4).

**Table 4:** Functional scores.

	Primary	Revision	*p* value
IKDC score	81.05 (71.7–89.7)	80.4 (67.85–83.85)	0.153
Lysholm score	95.0 (89.0–99.0)	92.5 (88.0–95.0)	0.324

IKDC: International Knee Documentation Committee.

Results are shown as median with interquartile range.

## Discussion

The principal findings of this study are patients with revision ACL reconstructed knees had a higher AP translation and a higher percentage of instability than those with primary ACLR surgery. However, after planned rehabilitation, patients in revision groups can have non-inferior results in the isokinetic knee strength and knee functional outcomes compared to those with primary ACLR surgery.

Revision ACLR surgeries are known to show inferior results compared with those for primary ACLR surgeries with respect to postoperative instability, return to sports, and patients’ satisfaction^2,10,14–16^. However, it is unclear that whether the poor clinical outcomes are from knee joint instability or other factors such as muscle strength. Gifstad *et al*. found an overall reduction in muscle strength for the injured knee compared with the uninjured knee in the patients with revised ACLR, which correspond to the decreased performance in various knee functional scores^10^. Meanwhile, several studies showed loss of knee flexion strength only^17–20^ or both flexion and extension muscle power in patients with the revision ACLR surgery but not in those with the primary ACLR surgery^10^. In the current study, though with higher degrees of joint instability, patients in the revision ACLR group still had similar performance in the knee flexion and extension muscle power, and had similar clinical outcomes when compared with those in the primary ACLR group.

The ideal graft for ACLR surgery remains controversial. Nevertheless, the degree of joint instability is reported to be associated with graft laxity, whereas allografts can theoretically trigger immune responses and show relatively slower postoperative reformation^21,22^. In a study comparing autografts and allografts in ACLR surgery, a significant difference of ≥3 mm was found in ligament laxity measurements in 14.9% of autografts and 31.1% of allografts^23^. In contrast, there was no difference in laxity between the revision group and primary group when only autologous hamstring tendon was used as the graft material^24^. Accordingly, in this study, we used double-looped semitendinosus and gracilis autografts for primary ACLR compared to tibialis anterior tendon allografts for revision ACLR. Considering significant laxity as side-to-side difference more than 5 mm in AP laxity^25,26^, none in primary group and five patients of the revision group (12.5%) showed laxity of ≥5 mm in this study. As such, we found a statistically significant difference in the joint laxity between two groups. To sum up, this difference between the two groups may possibly attribute to the different graft materials used.

Muscle strength is a factor greatly influencing knee function. Therefore, it is important to accurately determine the level of muscular strength during postoperative recovery. The quadriceps muscle plays a key role in maintaining dynamic stability of the knee joint, whereas hamstring muscles act as an agonist to the ACL preventing anterior dislocation of the knee^27^. Atrophy of these two muscles causes more functional instability and impairment. Although isokinetic equipment is widely used for accurate and objective assessment of muscle strength in the knee joint^1,10,11^, evidence on revision ACLR surgery using isokinetic equipment to assess muscle strength is lacking. In this study, we used isokinetic equipment to assess the degree of muscle strength recovery in the knee joint between the two groups at the same time point of 1 year postoperatively. The two groups had similar outcomes in isokinetic muscle strengths, which corresponded to similar results in functional scores.

Uribe *et al*.^28^ reported that the outcomes of revision ACLR, in that only about half patients returned to their pre-injury activity level which was not as good as primary ACLR, regardless of the type of graft materials. However, we observed similar results in extensor and flexor muscle defect rates between primary and revision ACLR groups. Moreover, there were also no differences in functional knee scores (IKDC and Lysholm score) between the two groups. That is, after planned training and rehabilitation, patients with revision ACR still can have good results in knee strength and functions.

Our study had four limitations. First, there is the small number of revision ACLR cases, and all patients are males. The findings of our study cannot apply to the female patients. Further studies on females are needed. Second, the fixation methods of tendon graft were not identical with and between two groups, which cause inherent bias of this study. Third, the pre-injury status of each patient was hardly recorded, which might make the interpretation of findings difficult, especially muscle strength. However, we presented the data of isokinetic muscle strength deficit by comparing the healthy limb, which could minimize this potential bias. Fourth, we cannot address the impact of potential covariates including commitment meniscus injury and critical physical findings status due to incomplete recording and limited sample size. Further study with a large sample will validate the findings of comparison after adjustment of potential covariates.

In conclusion, patients with revision ACLR surgery had significantly higher AP laxity than those with primary ACLR surgery but can have similar results in knee strength and functions after rehabilitation. More intensive rehabilitation is suggested for patients with revision ACLR surgery.

## Methods

### Subjects and demographics

This is a cross-sectional study with 80 male patients with arthroscopic ACL reconstruction surgery (40 primaries and 40 revisions) recruited from Samsung Medical Center between April 2013 and May 2016. Hamstring (semitendinosus-gracilis) tendon autografts were used in the primary ACL reconstruction group, while tibialis anterior tendon allografts were used in revision ACL reconstruction group. The grafts were fixed by a cortical suspensory device (ENDOBUTTON CL, Smith-Nephew, London, UK) for the femoral side and bioabsorbable interference screws with a post-tie for the tibial side. The exclusion criteria were that patient who had more than one revision ACL reconstruction surgery on the same knee, combined/multiple ligamentous injuries or previous surgery on the same lower limb. All participants provided informed consent prior to testing, and the study was approved by the Sungkyunkwan University School of Medicine Clinical Research Ethics Board (SMC 2016-09-074-001). The committee that approved the research, confirm that all research was performed in accordance with relevant guidelines/regulations. Informed consent was obtained from all participants and/or their legal guardians.

### Rehabilitation

The same postoperative rehabilitation program was used for primary and revision groups. At the first week right after the operation, the general light intensity of isometric exercises and range of motion (ROM) exercises were performed. The angle of knee flexion increased 15° each week in ROM exercise program. At the fourth week, the knee joint range of motion achieved at 90°, and reached 130° at the sixth week. The patients subjected to partial weight bearing for 4–6 weeks postoperatively and progressively switched to full weight-bearing. By the 6–12 weeks, the patient would achieve combined strength, endurance and balance exercise without pain. At six months, light running was allowed. By nine month after surgery, the patient could receive sports-related training if there were no problems such as effusion, pain, or knee instability.

## Assessment

Anteroposterior (AP) knee translation was assessed via the KT-2000 arthrometer (MEDmetric, San Diego, CA, USA) with maximal manual tension and a knee flexion angle of 20° and 30 lb (134 N) anterior force at 12 months after surgery. The measurements were done three times, and a mean value was calculated. These examinations were done by the same personnel who have several years of arthrometry experience to minimize the errors of measurements. Side-to-side AP laxity difference between injured and noninjured knee was also recorded.

At 12 months after index surgery, Isokinetic knee strength with peak extensor and flexor torque (Nm/kg) was evaluated via a CSMI dynamometer (CSMI Medical Solutions, Stoughton, MA, USA) whereby angular velocities of 60°/sec and 180°/sec were measured to determine isokinetic knee strength. After warming up on a cycle ergometer for 5 min, subjects were allowed to practice the test protocol for familiarization. Subsequently, patients allowed to perform the strength test 3 times with a 1 minute washout period between each test session. While doing the sessions, patients were encouraged to perform with the maximum effort by the examiner. Isokinetic concentric measurements of quadriceps and hamstring strength demonstrated excellent reliability^29^. Both injured and non-injured side were measured for three times, and a mean difference was calculated as

1 $$strength\,deficit=(noninjured\,limb-injured\,limb/noninjured\,limb)\ast 100 \% $$

Knee joint functional scores were evaluated at 12 months after index surgery using International Knee Documentation Committee (IKDC)^30^ and Lysholm knee scales^31^. The IKDC knee scale quantifies symptoms, sports activities, and function^30^. The Lysholm knee scale quantifies pain, instability, locking, swelling, stair-climbing, squatting abilities, and need for support^31^.

### Statistical analysis

A priori calculated power >0.80 at an alpha level equal to 0.05 was used to determine that a sample size of 35 for each group was necessary to determine in KT-2000 arthrometry value^32^. All data statistical analysis was performed using SPSS version 13.0 (SPSS Inc., Chicago, IL, USA). Chi-Squared test and Mann-Whitney U test were used throughout to compare variables between two groups, with *p* < 0.05 considered statistically significant. All data were reported with median and interquartile range (IQR).

### Data availability

The datasets generated during and analysed during the current study are not publicly available due to policy of the Sungkyunkwan University School of Medicine Clinical Research Ethics Board but are available from the corresponding author on reasonable request.
